# The “Extreme Exercise Hypothesis”: Recent Findings and Cardiovascular Health Implications

**DOI:** 10.1007/s11936-018-0674-3

**Published:** 2018-08-28

**Authors:** Thijs M. H. Eijsvogels, Paul D. Thompson, Barry A. Franklin

**Affiliations:** 1 Radboud Institute for Health Sciences, Department of Physiology (392), Radboud University Medical Center, P.O. Box 9101, 6500 HB Nijmegen, The Netherlands; 2 Division of Cardiology, Hartford Hospital, Hartford, CT USA; 3 Department of Cardiovascular Medicine, William Beaumont Hospital, Royal Oak, MI USA

**Keywords:** Endurance exercise, Atherosclerosis, Myocardial fibrosis, Sudden cardiac death, Physical activity, Athletes

## Abstract

**Purpose of review:**

The “Extreme Exercise Hypothesis” is characterized by a U-shaped or reverse J-shaped, dose-response curve between physical activity volumes and cardiovascular health outcomes. In this review, we summarize recent findings that may support or refute the “Extreme Exercise Hypothesis.” Furthermore, we discuss potential cardiovascular health implications of the cardiac anatomical, structural, contractility, and biomarker abnormalities that have been reported in some veteran endurance athletes.

**Recent findings:**

Emerging evidence from epidemiological studies and observations in cohorts of endurance athletes suggest that potentially adverse cardiovascular manifestations may occur following high-volume and/or high-intensity long-term exercise training, which may attenuate the health benefits of a physically active lifestyle. Accelerated coronary artery calcification, exercise-induced cardiac biomarker release, myocardial fibrosis, atrial fibrillation, and even higher risk of sudden cardiac death have been reported in athletes.

**Summary:**

There is primarily circumstantial evidence that supports the “Extreme Exercise Hypothesis.” Subclinical and atherosclerotic coronary artery disease (CAD) as well as structural cardiovascular abnormalities and arrhythmias are present in some of the most active veteran endurance athletes and need appropriate clinical follow-up to reduce the risk for adverse cardiovascular outcomes. Future studies are warranted to establish the long-term cardiovascular health effects of these findings in veteran endurance athletes.

## Introduction

The World Health Organization (WHO) recommends that adults aged 18–64 engage in at least 150 min/week of moderate intensity aerobic activities or 75 min/week of vigorous intensity aerobic activities or an equivalent combination thereof [1]. Furthermore, complementary muscle-strengthening activities should be performed involving major muscle groups on two or more days a week. Regular aerobic exercise and resistance training are associated with a reduced risk for cardiovascular morbidity [2, 3] and mortality [4, 5]. The dose-response relationship between aerobic exercise volumes, which largely reflect the duration and intensity of physical activity, and the associated health benefits is most often described as curvilinear, with the greatest exercise-induced health improvements at the beginning of the curve [6]. Nevertheless, the WHO recommends that more exercise is better, and it is estimated that maximal cardiovascular health benefits are obtained at an exercise volume that approximates up to ~ 3 to 4 times the current exercise recommendations [7, 8].

**Fig. 1:**
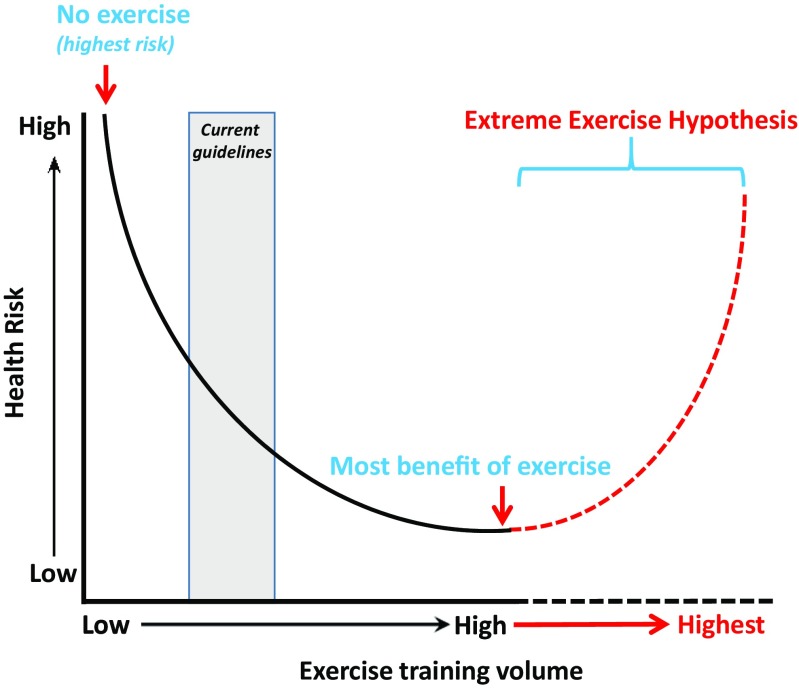
Conceptual overview of the “Extreme Exercise Hypothesis.” Increasing volumes of exercise lead to a curvilinear decrease in health risks, but these health benefits may be partially lost once an individual performs exercise training beyond the optimal exercise dose.

The health effects of exercise volumes beyond the “optimal dose” are currently under debate. Some epidemiological studies reported an increased risk of disease and/or mortality at the highest exercise volumes [4, 9••, 10], suggesting that health benefits of an active lifestyle may plateau or even decline in extreme exercisers [11]. Cross-sectional studies have reported that the most active veteran endurance runners have an increased risk for myocardial fibrosis [12, 13], coronary artery calcification [14•], and atrial fibrillation [15]. These observations imply that high volumes of chronic endurance exercise training may be detrimental for the heart. Hence, the “Extreme Exercise Hypothesis” suggests a revision of the dose-response association between physical activity volumes and health outcomes, which is characterized by a U-shaped or reverse J-shaped curve (Fig. 1).

Confirmation or rejection of the “Extreme Exercise Hypothesis” is a hot topic in the field of sports cardiology. There are valid concerns that the message that high-volume, high-intensity exercise can potentially harm the heart, may discourage a physically active lifestyle among the general population and contribute to the increasing prevalence of physical inactivity. The aims of this review are to (1) summarize recent findings that support or refute the “Extreme Exercise Hypothesis” and (2) interpret the potential effects of exercise-induced (mal)adaptations on cardiovascular health outcomes.

## Identification of a potential upper limit

Few studies had sufficient power to explore the dose-response relationship between physical activity and cardiovascular health outcomes, which allows identification of a potential upper limit. Arem et al. combined leisure-time physical activity data from six prospective population-based cohorts from the USA and Europe to create a study population of 661,137 individuals [9••]. Maximal risk reduction for all-cause mortality occurred at an exercise volume of 3 to 5 times current exercise recommendations (hazard ratio [HR]: 0.61, 95% confidence interval [CI]: 0.59–0.62). Individuals performing physical activity at a volume ≥ 10 times current exercise recommendations had a lower mortality risk (HR: 0.69, 95% CI: 0.59–0.78) compared to the inactive reference group, but health benefits in the most active group were smaller compared to the optimal exercise volume group (31% vs. 39% risk reduction). Lear et al. more recently analyzed a cohort of 130,843 individuals from 17 low- to high-income countries to explore the effect of physical activity on mortality and cardiovascular disease (CVD) [4]. Individuals in the high physical activity group had a substantially reduced risk for all-cause mortality (HR: 0.65, 95% CI: 0.68–0.77) and major CVD (HR: 0.75, 95% CI: 0.69–0.82) compared to the low physical activity group. Recreational physical activity at ~ 112 min/week yielded the largest risk reductions for the combined endpoint of mortality and major CVD (HR: 0.89, 95% CI: 0.82–0.95), but these significant health benefits were lost at physical activity volumes above 255 min/week. These observations challenge the notion that more exercise is invariably better. However, it remains difficult to differentiate between (1) a real finding that supports the “Extreme Exercise Hypothesis” and (2) a loss of health benefits due to statistical factors produced by the relatively small number of individuals in the most active group. This possibility is supported by the large confidence intervals for the risk estimates. Based on limited current evidence and numerous potential confounders, it is difficult to delineate an upper limit for the benefits of physical activity at this time. Future studies should combine data from large cohorts, including highly active amateur athletes to determine the health effects of the highest volumes of physical activity.

## Accelerated coronary artery calcification

Atherosclerotic coronary artery disease (CAD) is the largest cause of CVD and is highly dependent on both genetic and lifestyle factors [16]. Regular physical activity and exercise training are associated with a reduction of selected CVD risk factors, including lipid levels [17], blood pressure [18], and inflammation [19]. Coronary artery atherosclerosis can be assessed using different imaging techniques (Table 1), of which computed tomography (CT) is the least costly, fastest and most widely used. The coronary artery calcification (CAC) score can be derived from CT images and is an excellent predictor of future cardiac events [20]. Interestingly, Möhlenkamp et al. found a higher prevalence of CAC scores ≥ 100 Agatston units among 108 marathon runners (36%) compared to an age and risk factor-matched control group from the general population (22%).

**Table 1:** Imaging techniques to assess underlying atherosclerotic CAD and/or myocardial fibrosis in endurance athletes

Coronary artery atherosclerosis		Myocardial fibrosis	
Technique	Outcome	Technique	Outcome
CT	Coronary artery calcification (CAC) score	Transvenous endomyocardial biopsy—picrosirius red or Masson staining	Fibrillar collagen quantification
CT coronary angiography	Plaque characteristics (i.e., plaque phenotype, plaque volume)	Cardiac MRI with gadolinium contrast infusion	Presence and volume of focal myocardial fibrosis
IVUS	Atherosclerotic burden and plaque characteristics	Cardiac MRI with T1 mapping	Presence and volume of diffuse myocardial fibrosis
OCT	Plaque lipid content, macrophage infiltration and thickness of fibrous cap		
Cardiac MRI	Plaque characteristics		

*CT* computed tomography, *IVUS* intravascular ultrasound, *OCT* optical coherence tomography, *MRI* magnetic resonance imaging, *CAD* coronary artery disease

Recent studies have provided important novel insights regarding this widely cited observation. Aengevaeren et al. examined the association between lifelong volumes of physical activity and the prevalence and characteristics of coronary atherosclerosis in 284 male amateur athletes [21••]. The most active athletes routinely exercised at volumes equal to four times current recommendations, whereas the least active athletes exercised at the current recommended volume. The most active athletes had a higher CAC prevalence than the least active athletes (68% versus 43%, odds ratio [OR]: 3.2, 95% CI: 1.6–6.6). However, the most active athletes also had a lower prevalence of mixed plaques (48% versus 69%; OR: 0.35, 95% CI: 0.15–0.85) and more often had only calcified plaques (38% versus 16%; OR = 3.57; 95% CI: 1.28–9.97) compared with the least active athletes. This observation has important clinical relevance as mixed plaques are associated with a higher probability of future cardiovascular events compared with calcified plaques (38% versus 6%) [22]. Similar findings were reported in an English cohort of 152 veteran athletes and 92 sedentary controls [23••]. Male athletes more often had atherosclerotic plaques (44% versus 22%; *p* = 0.009) and a higher prevalence of CAC > 300 (11% versus 0%, *p* = 0.009) compared to age and risk factor-matched sedentary controls. Again, veteran athletes predominantly had calcified plaques, whereas mixed plaques were more prevalent among controls. In aggregate, these data suggest that long-term exercise training is associated with accelerated coronary artery atherosclerosis, but that accelerated plaque calcification may outweigh the cardiovascular risks associated with increased CAC scores. Additional longitudinal studies are needed to confirm this hypothesis.

Lin et al. assessed changes in plaque characteristics among eight participants of the Race Across the USA (140 race days covering 3080 miles) [24]. Four runners had no evidence of CAD on CT angiography before or after the race, but luminal stenosis and plaque volume (range: 4.8–94 mm^3^) increased in four runners with coronary atherosclerosis present before the race. The change in plaque volume was mainly attributed to an increase in non-calcified plaque. However, concomitant increases in high sensitivity C reactive protein (CRP) were noted, suggesting that exercise-induced inflammation may contribute to accelerated plaque progression. Whether initial increases in non-calcified plaque volume may change to calcified volume during race recovery is unknown. Previous studies demonstrated that exercise increased parathyroid hormone [25], decreased vitamin D3 [26] and decreased magnesium levels [27], all biomarkers of calcium-phosphate metabolism, which could affect vascular calcification [28]. Future studies investigating the underlying mechanisms of accelerated coronary artery atherosclerosis in veteran athletes are needed and may provide insight into how to improve strategies to stabilize plaques in vulnerable patient populations.

### Increased myocardial fibrosis

Exercise-induced increases in cardiac biomarkers, such as troponin, a marker of cardiomyocyte damage, and B-type natriuretic peptide (BNP), a marker of myocardial stress, are common in athletes after endurance exercise [29]. Recent studies explored the impact of endurance exercise on novel cardiac biomarkers, such as galectin-3, a marker of myocardial fibrosis, [30] and soluble suppression of tumorigenicity-2 (sST2), a marker of extracellular matrix remodeling and fibrosis [31]. Resting levels of galectin-3 were higher in athletes (*n* = 21) compared to controls (*n* = 21), whereas significant increases were observed following a 30-km run (12.8 ± 3.4 to 19.9 ± 3.9 ng/ml, *p* < 0.001) [30]. Similarly, sST2 concentrations increased following a marathon (34.2 to 54.2 ng/ml, *p* < 0.001), with 68 of 79 athletes (86%) demonstrating a concentration above the upper reference limit. Complete normalization of sST2 levels occurred within 48 h [31]. Increases in cardiac biomarkers are modest and transient, but the clinical implications of these elevations are unknown. Accordingly, long-term exercise training/competition with repetitive exposure to prolonged vigorous exercise may increase cardiac fibrosis.

Cardiomyocyte damage leads to myocardial fibrosis, which is characterized by collagen infiltration in the extracellular matrix. The presence and magnitude of myocardial fibrosis can be determined via microscopic analysis of cardiac muscle obtained by postmortem biopsy or via cardiac magnetic resonance imaging (MRI; Table 1). Previous studies using MRI in athletes reported that the prevalence of myocardial fibrosis varied substantially (0% to 50%) between study populations [13, 32]. A systematic review found evidence of myocardial fibrosis in 30 of 509 scanned athletes (5.9%) [33•]. Myocardial fibrosis patterns were heterogeneous, and most frequently located near the interventricular septum and the right ventricular insertion points. The presence of myocardial fibrosis was strongly associated with the cumulative exercise dose. In contrast to studies from Germany [34] and the USA [35]. Bohm et al. found no difference in left and right ventricular function parameters between 33 competitive elite male master endurance athletes and 33 controls matched for age, height, and weight, [34], and myocardial fibrosis was observed in only 1 athlete. Abdullah et al. compared left ventricular characteristics across groups of long-term exercisers (< 2/2–3/4–5/6–7 exercise sessions/week) and found a stepwise improvement of cardiac structure and function with increasing doses of physical activity [35]. Among the 92 study participants, delayed gadolinium enhancement was observed in only 1 exerciser in the 2–3 sessions/week group. These studies suggest that myocardial fibrosis is a rare finding among endurance athletes. The discrepancy in prevalence rates may be due to the age and training status of the study population or to survival bias. On the other hand, Wilson et al. used delayed gadolinium enhancement on cardiovascular MRI to describe diverse patterns of myocardial fibrosis in 6 of 12 highly trained veteran endurance athletes [36]. Additional prospective studies are needed to confirm the association between exercise dose and incident myocardial fibrosis.

Whereas previous studies predominantly employed gadolinium infusion to determine focal fibrosis, T1 mapping is increasingly used to quantitate diffuse fibrosis (Table 1). Gormeli et al. found that athletes had significantly higher left ventricular (LV) native T1 values (1230 ± 39 ms versus 1174 ± 36 ms, *p* < 0.001) and interventricular septum (IVS) native T1 values (1268 ± 48 ms versus 1180 ± 27 ms, *p* < 0.001) compared to matched sedentary controls [37]. Furthermore, native T1 values of the LV (*p* < 0.05) and IVS (*p* < 0.05) were significantly higher in athletes that trained ≥ 5 years as compared to those training < 5 years, and the highest values of LV end diastolic volume and IVS wall thickness were found in those athletes who had trained the longest. These data suggest that more training results in greater cardiac remodeling, but potentially also more diffuse myocardial fibrosis.

The clinical consequences of myocardial fibrosis in athletes are largely unexplored. A German study found that coronary revascularization was more common in athletes with, than without, fibrosis, 25% versus 1%, respectively [12]. Schnell et al. reported a case series of serious cardiac complications in Belgian athletes with isolated subepicardial fibrosis, such as non-sustained ventricular arrhythmias, symptomatic ventricular tachycardia, and progressive LV dysfunction [34]. British veteran athletes with myocardial fibrosis demonstrated normal cardiac function, but co-localized regional cardiac dysfunction was found in fibrotic areas, substantiated by evidence of an attenuated cardiac strain and base to apex gradient [38]. These observations suggest that the presence of myocardial fibrosis requires appropriate clinical follow-up to evaluate the possibility of future adverse cardiovascular outcomes.

## Exercise and atrial fibrillation

**Fig. 2:**
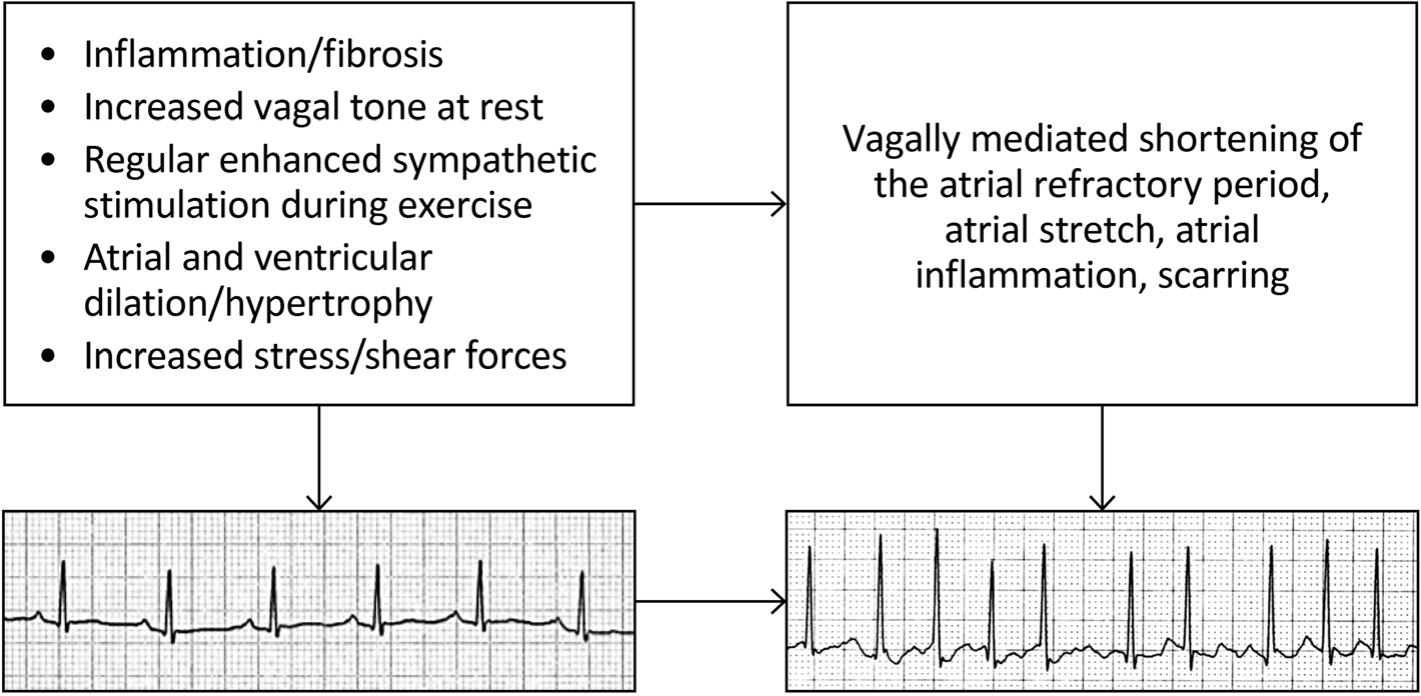
Potential mechanisms and associated sequelae for atrial fibrillation induced by strenuous endurance exercise. The combination of autonomic, structural, and hemodynamic effects of high-volume, high-intensity aerobic exercise, repeated over time, likely impart some of the increased risk for arrhythmias (Adapted from [7, 46–48]).

The association between habitual physical activity, cardiorespiratory fitness (CRF), expressed as mL O_2_/kg/min or metabolic equivalents (METs; 1 MET = 3.5 mL/kg/min), and incident atrial fibrillation (AF) is complex. Two recent studies reported that higher CRF was associated with a graded reduction in the risk of AF [39, 40]. This observation is in contrast to a prospective observational study in older adults [41] and a large cohort study of long-distance cross-country skiers [42••] that found that individuals participating at the highest intensities and/or volumes of exercise were at greater risk of developing AF. Another systematic review and meta-analysis of case-control studies found that the overall risk of AF was significantly higher in athletes than controls, with a 5.29 OR (95% CI: 3.57–7.85; *p* = 0.0001) [43]. Others have reported that practicing endurance sports increases the probability of experiencing AF by two- to tenfold, even after adjusting for potential confounding variables and associated risk factors [44], and that the life-time-accumulated hours of vigorous endurance training, specifically ≥ 2000 h, was the most powerful predictor of exercise-induced AF [45]. Potential mechanisms for AF induced by long-term strenuous endurance exercise are shown in Fig. 2 [7, 46–48]. The combination of autonomic, structural, and hemodynamic effects of high-volume, high-intensity aerobic exercise, repeated over time, probably contribute to the increased risk for AF. Additional pathophysiologic mechanisms may include derangements in sympathetic/parasympathetic tone and recurrent fluid and electrolyte shifts.

On the other hand, for the general population, increased CRF is associated with a reduced risk of AF [39, 40]. There is also at least one non-randomized study demonstrating that increases in CRF achieved by a physician-led exercise program reduces the recurrence of AF in obese patients [49], even when weight loss is minimal [50]. These findings suggest that the relationship between physical activity and incident AF is best summarized by a reverse J-shaped curve. Light-to-moderate amounts of exercise decrease, but larger volumes of exercise potentially increasing the risk of AF [7].

## Sudden cardiac death

High-intensity exercise can acutely, albeit transiently, increase the risk for sudden cardiac arrest (SCA) or sudden cardiac death (SCD) in individuals with underlying cardiac disease [51]. The cause of death is usually different for young (≤ 40 years) versus older athletes (> 40 years). Young individuals die during exercise primarily from inherited or congenital cardiac conditions, such as hypertrophic cardiomyopathy (HCM), coronary artery anomalies, and right ventricular cardiomyopathy (RVCM) [52], whereas atherosclerotic coronary artery disease (ASCAD) is the primary cause of death in older athletes [53]. A Canadian study of athletic participants aged 12–45 years old found 74 cases with SCA over the course of 18.5 million persons-years of observation, yielding an incidence of 0.76 cases per 100,000 athletes per year [54••]. A total of 16 SCA cases occurred during competitive sports of which 44% survived, whereas 58 cases occurred during non-competitive sports of which 44% also survived. More importantly, genetic structural abnormalities, such as HCM and RVCM were uncommon causes of SCA (8% and 5%, respectively). This is in contrast to some primarily older studies, which identified HCM as the predominant cause of SCD in young athletes [52]. Early risk identification of gene carriers for conditions, such as HCM and RVCM and subsequent exercise restriction, may have contributed to the apparent change in the cause of SCD in young athletes [55]. Another study from Australia and New Zealand found a similar incidence of SCD (1.3 cases per 100,000 persons/year) in children and young adults [56], but included all deaths and not just exercise-related deaths. SCD incidence rate increased with age and was highest for individuals aged 31–35 years (3.2 cases per 100,000 persons/year). The Australian study reported that most SCD cases occurred during sleep (28%) and rest (20%) and relatively few cases occurred during light physical activity (14%), exercise (8%), or post-exercise (3%) [56]. However, the investigators did not correct SCD incidence for exposure time, which precludes an accurate assessment of the relative risks of exercise. Since most people spend more time asleep than in physical activity, the 25% incidence of SCD during all intensities of exercise suggest that exercise increases the risk of SCD compared to non-exercise activities. Nonetheless, the exercise-induced risk for SCA and SCD is only transient, and there is strong evidence that regular exercise training is associated with an overall decreased risk of adverse cardiovascular outcomes [57•].

## Conclusions

Recent studies demonstrated that extreme volumes and/or intensities of long-term exercise training are associated with several possible cardiac maladaptations. First, some epidemiological studies have reported an increased risk for adverse cardiovascular outcomes at the upper end of the physical activity spectrum. Nevertheless, there is no clear threshold for an upper limit of the exercise-induced health benefits. Second, the most active older athletes often demonstrate a higher coronary artery calcification score, but atherosclerotic plaques are more likely calcified which reduces the risk of plaque rupture. The associated cardiovascular risk implications of these observations are currently unknown. Third, elevations of biomarkers for cardiomyocyte damage and myocardial fibrosis are common following intense exercise but normalize soon after exercise cessation. Focal and diffuse fibrosis is found in a small subgroup of veteran athletes, but again, the significance of this finding is unknown. Fourth, a reversed J-shaped association is found between exercise volumes and atrial fibrillation, with a reduced risk at light-to-moderate volumes and an increased risk at high volumes. Fifth, SCA and SCD are infrequent among exercising young individuals, with an estimated incidence rate of 0.76 per 100,000 person years. Collectively, these data suggest: (1) there is limited evidence that supports the “Extreme exercise hypothesis,” the most compelling relating to the increased risk of atrial fibrillation at high volumes of exercise; (2) cardiac anomalies may be present in a small proportion of the most active veteran athletes; and (3) the combination of high-intensity physical activity in the presence of known or occult CVD, seems to be the major cause of exercise-related fatalities.
